# Current practice, training and skill assessment of central venous access device insertion: Perspectives of intensive care trainees in adult intensive care units across Australia and New Zealand and their recommendations for improvement: **C**entral Venous **L**ines **I**nsertion: **P**ractice, **E**ducation, **R**ecommendation 2.0 **(CLIPER 2.0)**

**DOI:** 10.1016/j.ccrj.2025.100159

**Published:** 2026-02-19

**Authors:** Tapan Parikh, Wisam Al Bassam, Yahya Shehabi, Deepak Bhonagiri, Tim Leong, Adrian Pakavakis, Brendan Murfin, Ashwin Subramaniam

**Affiliations:** a Intensive Care Unit, Monash Health, Australia; b Intensive Care Unit, Liverpool Hospital, Australia; c Monash University, Peninsula Clinical School, Australia; d Macquarie University, Australia; e Western Sydney University, Australia; f University of New South Wales (UNSW), Australia; g Monash University, Australian and New Zealand Intensive Care Research Centre, School of Public Health and Preventive Medicine, Australia

**Keywords:** CVAD, CVC, Central venous line, Training, ANZ, Accreditation, Survey, Practice, Recommendations

## Abstract

**Objective:**

Central venous access device (CVAD) insertion is a routine procedure in intensive care units (ICUs); however, it is associated with procedural risks. While structured training enhances safety, significant variability exists in training, supervision, and competency assessment across ICUs. Standardised education and assessment frameworks are recommended to improve procedural safety and patient outcomes. This study aimed to evaluate ICU trainees’ experiences with CVAD insertion training, identify any significant variation in current educational frameworks, and gather recommendations for enhancing training and assessment.

**Design:**

A web-based survey was distributed to ICU trainees across Australia, New Zealand, Singapore, and Hong Kong. Data were analysed using descriptive statistics and regression models.

**Main outcome measures:**

Key outcomes included trainee's perceptions of training disparities, accreditation processes, and practice variations across ICUs, informing the development of a standardised CVAD training framework.

**Results:**

Among 237 respondents, 199 responses were analysed. Fewer than two-thirds of trainees in tertiary and metropolitan ICUs and only 17% in regional and private ICUs, reported access to structured multimodal CVAD training, while 15.3% indicated no formal training was available. Fewer than a quarter (23.1%) of less experienced trainees reported having undergone competency assessments in the past 12 months. Commonly perceived challenges included coordinating ultrasound guidance and manipulating the guidewire and catheter. Most trainees (52.3%) recommended six to ten supervised insertions and competency across multiple insertion sites (∼60%) for accreditation.

**Conclusions:**

These findings highlight trainees' perceptions of critical gaps in CVAD training, emphasising the need for structured multimodal education, standardised competency assessments, and improved access to training resources across diverse ICU settings.

## 1 Introduction

Central venous access device (CVAD) insertions are common in intensive care units (ICUs). They are required in approximately 75% of critically ill patients for drug administration, haemodynamic monitoring, parenteral nutrition, and blood sampling.^1^ Despite being a routine procedure, CVAD insertion carries inherent risks and exhibits considerable variability in practitioner competency, training, and supervision. The CLIPER study, conducted among intensive care specialists across Australia and New Zealand (ANZ), highlighted significant discrepancies in training, skill assessment, accreditation, and supervision for CVAD insertion in adult ICUs.^2^

Competency-based education, including didactic teaching on anatomy and ultrasound physics, simulation training, and structured assessments, is recommended for safe CVAD insertions.^3^ Ultrasound guidance improves insertion success and reduces complications, thereby improving procedural safety.^4^ However, training resources and opportunities differ significantly across ICUs. Importantly, ICU trainees are primarily responsible for CVAD insertion and management, emphasising the need for a standardised training and assessment framework.

Following the CLIPER study’s findings on current practices, training, and assessment methods, it is essential to capture trainees' perspectives as they are the primary performers of these procedures. Therefore, we surveyed ICU trainees across ANZ to evaluate their experiences, identify gaps in training, and gather recommendations, which can be then used with Kern's Six-Step model^5^ and the Competency-Based Medical Education Framework^6^ for systematic curriculum development and competency alignment to improve CVAD training, assessment, and practice across ICUs.

## 2 Methods

A web-based questionnaire was developed to survey ICU trainees in adult ICUs across Australia, New Zealand, and College of Intensive Care Medicine (CICM)-accredited ICUs in Singapore and Hong Kong. The questionnaire was designed following a literature review, refined with input from clinical experts, and piloted before finalisation. The anonymous survey was hosted on the SurveyMonkey™ platform (Momentive Inc., San Mateo, California, USA), with details available in Supplementary Appendix 1.

The survey collected demographic information on ICU trainees and explored three key areas:^1^ current CVAD insertion practices,^2^ training and accreditation, and^3^ recommendations for future improvements.

### 2.1 Survey distribution and endorsements

The study was endorsed by the Australian and New Zealand Intensive Care Society and supported by the CICM. The Australian and New Zealand Intensive Care Society distributed the survey link to its members, requesting further dissemination to ICU trainees. A reminder email was sent after two months, and the survey remained open for three months. Additionally, the survey was disseminated to approximately 1100 ICU trainees via the CICM e-newsletter. To maximise participation, the survey was advertised on the CICM website and included in its monthly e-newsletters until January 31, 2024. Responses were collected until February 15, 2024, and consent was implied by survey completion.

### 2.2 Definition of CVAD

For this study, CVADs were defined as short-term, nontunnelled central venous catheters used for haemodynamic monitoring, vasoactive agent administration, and parenteral nutrition. To maintain consistency, peripherally inserted central catheters and sheath introducers were excluded.

### 2.3 Data collection

While 1100 trainees were officially registered with CICM, the survey was also distributed to nonregistered trainees working in ICUs. SurveyMonkey™ collected demographic data, including age, gender, country, state, years of experience, training stage, ICU type, and recency of ICU experience. Responses to all study questions were recorded anonymously.

### 2.4 Study outcomes

The primary outcome was to identify variation in CVAD insertion practice, training, and assessment from the perspective of ICU trainees. Secondary outcomes included trainees’ insights into training and accreditation, as well as recommendations for improvement.

### 2.5 Statistical analysis

Survey responses were reported as percentages of valid responses. Normally distributed data were presented as mean ± standard deviation, while non-normally distributed data were summarised using median and interquartile range. Bar charts were used to visualise trends. CVAD practice, training, and accreditation were analysed based on trainee experience (<3 years vs. ≥3 years of working in any ICU), with comparisons made using Chi-square tests. The three-year mark in our study was chosen pragmatically to reflect the typical duration over which ICU registrars gain sufficient exposure and assessment to be considered experienced. Multivariable ordinal logistic regression was conducted to identify factors associated with receiving CVAD insertion training in the past 12 months, reported as odds ratio with 95% confidence intervals (95% CI). To examine practice variations, two sensitivity analyses were performed: one stratified by primary workplace (public tertiary, metropolitan, rural/regional, or private ICU) and another by jurisdiction (Victoria, New South Wales, and other regions). All analyses were conducted as complete-case analyses using IBM SPSS Version 29 (Armonk, NY), with statistical significance set at p < 0.05. Free-text responses on training recommendations were analysed using “applied thematic analysis” and categorised by trainee experience level. The study used applied thematic analysis to analyse free-text responses on training recommendations, employing inductive, codebook-driven coding and systematic theme development. Credibility was strengthened through member checking; dependability and confirmability were supported by an audit trail and reflexive documentation. Thick description of context enhances transferability, enabling readers to assess applicability across settings.^7^

### 2.6 Ethics approval

This study was reviewed and approved by the Monash Health Research Ethics Committee (RES-23-0000-704QQ), with a waiver for informed consent.

## 3 Results

### 3.1 Study respondents

**Fig. 1:**
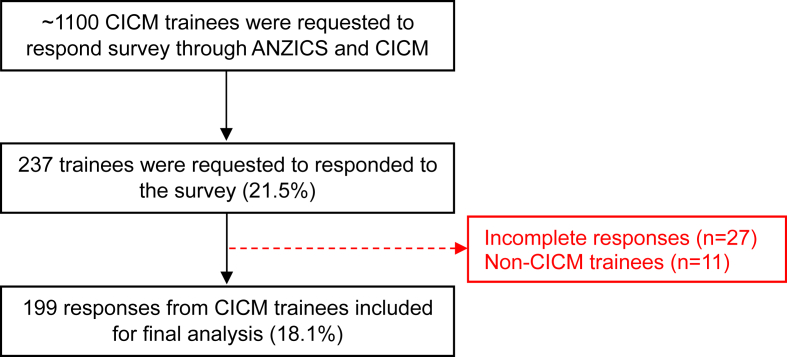
Consort flow diagram.

A total of 237 trainees (∼21%) working in ICUs responded to the survey. Of these, responses from 199 (18.1%) trainees affiliated with the CICM were analysed (Fig. 1). Most respondents were male (60%; n = 119), and 93.5% were from Australia, with the highest proportion from Victoria (44.2%; n = 88), followed by New South Wales (26.6%; n = 53) and Queensland (11.6 %; n = 23). More than half of the respondents (57.3%; n = 114) were between the ages of 30 and 40 years. Approximately 38.6% had less than three years of ICU experience.

Most respondents worked primarily in a tertiary ICU (71.4%), while a smaller proportion were based in metropolitan (17.1%), regional/rural (8.5%), and private (3.0%) ICUs. Regarding training levels, 37.2% of respondents were preprimary examination trainees, 34.7% had completed the primary examination, 14.6% were postfellowship examination trainees, and 13.5% were unaccredited trainees. Almost two-thirds (64.8%) had been working in ICUs for over 12 months. Detailed demographic characteristics are presented in Table 1.

**Table 1:** Demographics of respondents.

	Overall n = 199	Experience <3 years n = 77	Experience ≥3 years n = 122	p value
**Age**				**<0.001**
- <25 years	2 (1.0%)	2 (2.6 %)	0 (0)	
- 25–30 years	59 (29.6%)	49 (63.6%)	10 (8.2%)	
- 30–40 years	114 (57.3%)	26 (33.8%)	88 (72.1%)	
- >40 years	24 (12.0%)	0 (0)	24 (19.7%)	
**Gender**				**0.001**
- Male	119 (59.8%)	34 (44.2%)	85 (69.7%)	
- Female	79 (39.7%)	42 (54.5%)	37 (30.3%)	
- Nonbinary	1 (0.5%)	1 (1.3%)	0 (0)	
**Country of work**				0.88
**Australia**	**186 (93.5%)**	**72 (93.5%)**	**114 (93.4%)**	
- Australian capital territory	2 (1.0%)	1 (1.3%)	1 (0.8%)	
- New South Wales	53 (26.6%)	26 (33.8%)	27 (22.1%)	
- Northern territory	1 (0.5%)	0 (0)	1 (0.8%)	
- Queensland	23 (11.6%)	9 (11.7%)	14 (11.5%)	
- South Australia	9 (4.5%)	3 (3.9%)	6 (4.9%)	
- Tasmania	5 (2.5%)	1 (1.3%)	4 (3.3%)	
- Victoria	88 (44.2%)	31 (40.3%)	57 (46.7%)	
- Western Australia	6 (3.0%)	2 (2.6%)	4 (3.3%)	
**New Zealand**	**12 (6.0%)**	**4 (5.2%)**	**8 (6.6%)**	
- North Island	5 (2.5%)	2 (2.6%)	3 (2.5%)	
- South Island	6 (3.0%)	2 (2.6%)	4 (3.3%)	
**Other (Singapore)**	**1 (0.5%)**	**0 (0)**	**1 (1.3%)**	
**Hospital type**				**0.019**
- Tertiary	142 (71.4%)	49 (63.6%)	93 (76.2%)	
- Metropolitan	34 (17.1%)	15 (19.5%)	19 (15.6%)	
- Rural/regional	17 (8.5%)	12 (15.6%)	5 (4.1%)	
- Private	6 (3.0%)	1 (1.3%)	5 (4.1%)	
**>12 months experience in emergency department and/or anaesthetics**	145 (72.9%)	41 (53.2%)	104 (85.2%)	**<0.001**
**type of trainee**				**<0.001**
- Preprimary exam	74 (37.2%)	53 (68.8%)	21 (17.2%)	
- Postprimary exam	69 (34.7%)	13 (16.9%)	56 (45.9%)	
- Postfellowship exam	29 (14.6%)	0 (0)	29 (23.8%)	
- Unaccredited ICU registrar/CMO	27 (13.5%)	11 (14.3%)	16 (13.2%)	
**Years of experience**				**<0.001**
- 0 to 3 months	2 (1.0%)	2 (2.6%)	0 (0)	
- 3 to 6 months	0 (0)	0 (0)	0 (0)	
- 6 months to 1 year	16 (8.0%)	16 (20.8%)	0 (0)	
- 1 to 3 years	59 (29.6%)	59 (76.6%)	0 (0)	
- 3 to 5 years	51 (25.6%)	0 (0)	51 (41.8%)	
- 5 to 10 years	52 (42.6%)	0 (0)	52 (42.6%)	
- >10 years	19 (9.5%)	0 (0)	19 (15.6%)	
**Working continuously in ICU past 12 months**^a^	129 (64.8%)	42 (54.5%)	87 (71.3%)	0.52
**Commenced work in ICU within <12 months**^a^				0.27
- ≤6 months	30 (42.9%)	20 (66.7%)	10 (33.3%)	
- 6–12 months	40 (57.1%)	27 (67.5%)	13 (32.5%)	

CMO – career medical officer, ICU – intensive care unit.

a The trainee's experience at their current ICU site.

### 3.2 Training and accreditation for CVAD insertion skills

**Fig. 2:**
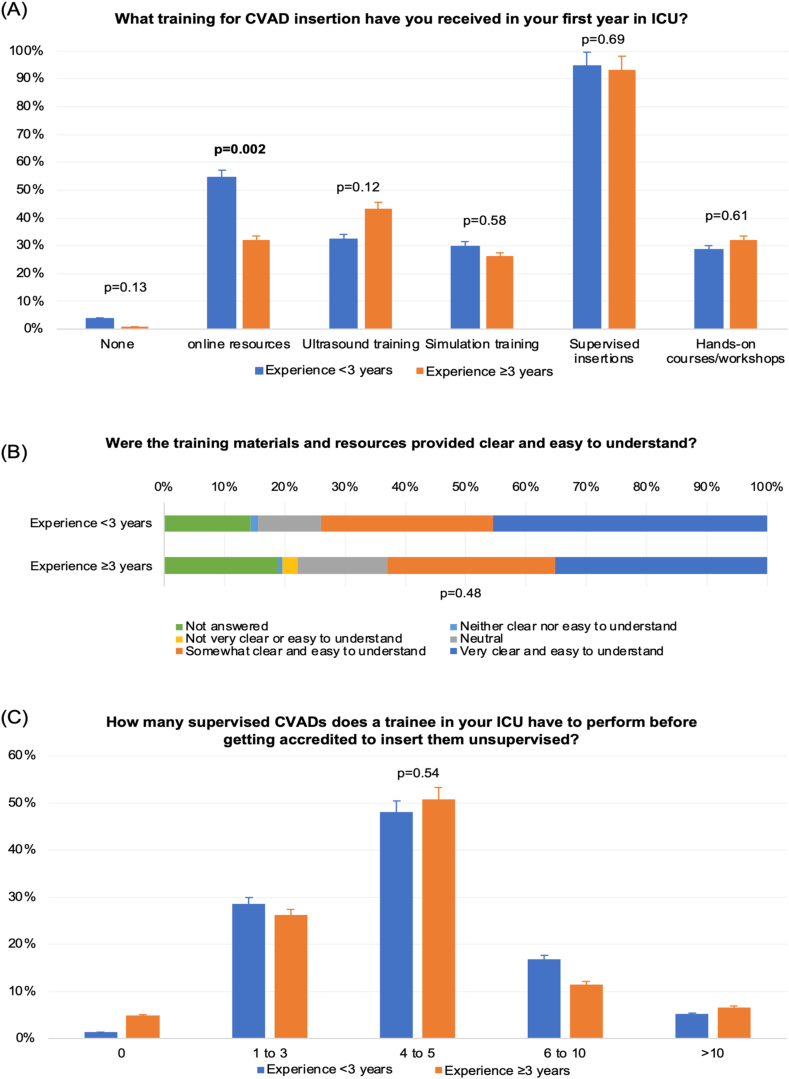
Training and accreditation for CVAD insertion skills. CVAD, central venous access device,

A summary of overall training experience and comparisons based on trainee experience levels are presented in Table 2 and Fig. 2. Over 90% of respondents reported that their CVAD insertion training involved supervised insertions, with no significant differences in group of <3 and >3 years of experience (Fig. 2A). Most respondents found the training materials and resources clear and easy to understand, with comparable responses between the two groups (Fig. 2B). Approximately half of the trainees indicated they inserted four to five CVADs before receiving accreditation to perform unsupervised insertions, while 15% inserted six to ten CVADs.

**Table 2:** Training experience and recommendations for training and accreditation.

		Overall	Experience <3 years	Experience ≥3 years	p value
**Training experienc**e					
Training received for CVAD insertion in the last 12 months?	No	134 (67.3%)	33 (42.9%)	101 (82.8%)	**<0.001**
	Yes	65 (32.7%)	44 (57.1%)	21 (17.1%)	
Does your current ICU have structured training that includes combinations of multimodal methods such as online/paper-based training, simulation, USG, and supervised practice?	I don't know	27 (13.6%)	13 (16.9%)	14 (11.5%)	0.52
	No	64 (32.2%)	25 (32.5%)	39 (32.0%)	
	Yes	108 (54.3%)	39 (50.6%)	69 (56.6%)	
If the answer was “No” or “I don't know” to the previous question, does your ICU have individual formal USG/simulation training, or supervised practice?	Neither	19 (15.3%)	5 (9.6%)	14 (19.4%)	0.05
	Only simulation	1 (0.8%)	1 (1.9%)	0 (0)	
	Only supervision	43 (34.6%)	24 (46.2%)	19 (26.4%)	
	Only USG	5 (4.0%)	3 (5.8%)	2 (2.8%)	
	Simulation + supervision	15 (12.1%)	7 (13.5%)	8 (10.9%)	
	USG + simulation	3 (2.4%)	2 (3.8%)	1 (1.4%)	
	USG + supervision	38 (30.6%)	10 (19.2%)	28 (38.9%)	
Do you use a checklist for CVAD insertion?	No	113 (56.8%)	37 (48.1%)	76 (62.3%)	0.10
	Not sure	9 (4.5%)	3 (3.9%)	6 (4.9%)	
	Yes	77 (38.7%)	37 (48.1%)	40 (32.8%)	
Does your supervisor for CVAD insertion use a checklist to assess you?	No	105 (52.8%)	42 (54.5%)	63 (51.6%)	0.51
	Not sure	39 (19.6%)	12 (15.6%)	27 (22.1%)	
	Yes	55 (27.6%)	23 (29.9%)	32 (26.2%)	
Do you feel that you were provided reasonable feedback by your supervisor who assessed your CVAD insertion skills?	No	39 (19.6%)	13 (16.9%)	26 (21.3%)	0.62
	Not sure	18 (9.0%)	6 (7.8%)	12 (9.8%)	
	Yes	142 (71.4%)	58 (75.3%)	84 (68.9%)	
Have you undergone an assessment of check for competency of your CVAD insertion skills in the last 12 months	No	153 (76.9%)	55 (71.4%)	98 (80.3%)	0.15
	Yes	46 (23.1%)	22 (28.6%)	24 (19.7%)	
Do you train junior doctors in inserting CVADs?	No	33 (16.6%)	28 (36.4%)	5 (4.1%)	<0.001
	Yes	166 (83.4%)	49 (63.6%)	117 (95.9%)	
**Trainee accreditation recommendation**					
How many CVADs should be supervised for novice trainees for accreditation in ICU?	<5	63 (31.7%)	26 (33.8%)	37 (30.3%)	0.22
	6–10	104 (52.3%)	43 (55.8%)	61 (50.0%)	
	>10	32 (16.1%)	8 (10.4%)	24 (19.7%)	
How many CVAD sites should be supervised for novice trainees for accreditation in ICU?	Single site	3 (1.5%)	1 (1.3%)	2 (1.6%)	0.50
	Multi-site	119 (59.8%)	50 (64.9%)	69 (56.6%)	
	Not answered	77 (38.7%)	26 (33.8%)	51 (41.8%)	
How should the competency for maintenance of CVAD insertion skills be assessed? (multiple responses answered)	Supervision of CVAD insertion + USG technique	171 (85.9%)	66 (85.7%)	105 (86.1%)	0.95
	Assessment by simulation	34 (17.1%)	13 (16.9%)	21 (17.2%)	0.95
	Online assessment only	37 (18.6%)	15 (19.5%)	22 (18.0%)	0.80

CVAD – central venous access device, ICU – intensive care unit, USG – ultrasound guidance.

Two-thirds of the respondents (67.3%) had not received CVAD training in the past 12 months, with significantly higher rates among those with ≥3 years of experience compared to those with <3 years (82.8% vs. 42.9%; p < 0.001) (Table 3). Less than two-thirds of the respondents from tertiary and regional ICUs, and only 17% of trainees from regional and private ICUs believed their primary workplace offered structured multimodal CVAD training, with no difference between the experience groups. Among those who indicated the absence of structured multimodal training in their ICUs, 30.6% reported having access to a combination of ultrasound training and supervised practice, while 15.3% stated that no training was available.

**Table 3:** Factors that relate to receiving any CVAD insertion training in the last 12 months.

Predictors	OR (95% CI)	p value
**Level of experience**		
- ≥3 years	Reference value	
- <3 years	6.56 (3.26–13.17)	**<0.001**
**Hospital type**		
- Metropolitan	Reference value	
- Private	0.44 (0.04–5.07)	0.51
- Rural/regional	0.28 (0.07–1.15)	0.08
- Tertiary	0.68 (0.28–1.66)	0.40
**Jurisdiction**		
- New South Wales	Reference value	
- Victoria	0.45 (0.20–1.03)	0.06
- Other^a^	0.70 (0.29–1.67)	0.42
**Working continuously in ICU past 12 months**	0.64 (0.32–1.28)	0.21

a Other = Other Australian States (except Victoria and New South Wales), New Zealand and Singapore. CVAD – central venous access device, ICU – intensive care unit, OR – odd ratio.

Not surprisingly, trainees with ≥3 years of experience more frequently trained junior doctors in CVAD insertion (95.9% vs. 63.6%; p < 0.001, Table 2). About 40% of trainees reported using checklists for CVAD insertion, while only 27.6% said that their supervisors used checklists for supervision. Both practices were comparable between experience groups. Furthermore, less than a quarter (23.1%) of respondents underwent competency assessments for CVAD insertion skills in the past 12 months, with no significant differences between the two groups. More than 70% of respondents reported receiving reasonable feedback from supervisors following CVAD insertions, again with no significant difference between groups.

### 3.3 CVAD insertion practice

**Fig. 3:**
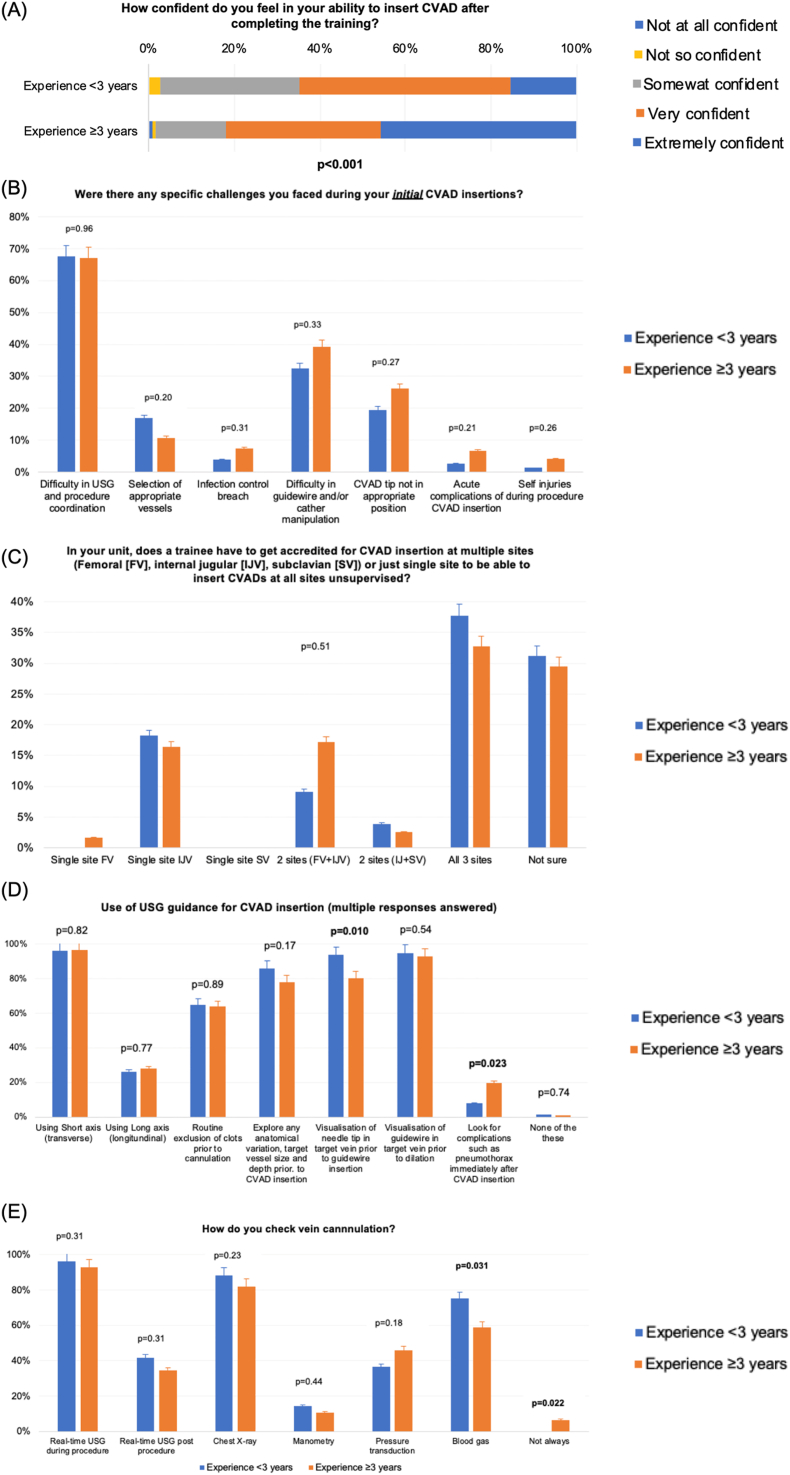
Trainee experience on the CVAD site and number needed to be supervised and methods to confirm CVAD placement. CVAD, central venous access device.

Trainees with ≥3 years of experience reported feeling significantly more confident in inserting a CVAD after completing training (p < 0.001; Fig. 3A). However, during initial CVAD insertions, the most frequently reported challenges included coordinating ultrasound guidance with the procedure, as well as manipulating the guidewire and catheter. Approximately one-quarter of trainees experienced difficulty in positioning the CVAD tip satisfactorily. Reassuringly, infection control breaches, acute complications, and self-injuries during the procedure were uncommon, with no significant differences between experience groups (Fig. 3B).

Most trainees indicated that competency in all three insertion sites (femoral, internal jugular, and subclavian veins) was required before performing unsupervised CVAD insertions (Fig. 3C). All trainees reported using ultrasound for CVAD insertion, with a preference for the short-axis approach (Fig. 3D). Most respondents (64 %) routinely excluded clots in veins using ultrasound, and 93 % visualised the needle tip before inserting the guidewire and confirming guidewire placement prior to dilatation. Less experienced trainees were more likely to visualise the needle tip before guidewire insertion (p = 0.010), while more experienced trainees were more likely to check for complications such as pneumothorax immediately after insertion (p = 0.023). No other significant differences in ultrasound use were observed between the groups.

More than 90% of trainees confirmed CVAD placement in a vein using real-time ultrasound, while over 80% used chest X-ray confirmation. Less experienced trainees more frequently utilised blood gas measurement to confirm venous placement compared to more experienced trainees (p = 0.031, Fig. 3E). Logistic regression analysis demonstrated that trainees with <3 years of experience were significantly more likely to have received CVAD insertion training in the past 12 months (Odds ratio = 6.56, 95% CI: 3.26–13.17; p < 0.001).

### 3.4 Sensitivity analyses

Sensitivity analyses based on ICU type (public tertiary, metropolitan, rural/regional, or private) and jurisdiction (Victoria, New South Wales, and other regions grouped together) demonstrated comparable results (Supplementary Tables 1-4, Supplementary Figures 1-4).

### 3.5 Recommendations from trainees

More than half the respondents (52.3%) recommended six to ten supervised CVAD insertions and competency in multiple insertion sites (∼60%) for accreditation of novice trainees. These recommendations were comparable across experience levels. A large proportion (86%) supported a combined approach involving direct supervision of CVAD insertions along with assessments of ultrasound technique to evaluate competency. Online (18.6%) and simulation-based (17.1%) assessments for skill competency were less preferred.

### 3.6 Free-text comments

Free-text responses from 44 trainees were thematically analysed and summarised in Table 4. Common themes included advocacy for online and ultrasound training with specific learning objectives (11%), targeted supervision in both clinical and simulated scenarios (22.7%), ongoing practice with regular competency assessments, and the use of checklists for supervisors and trainees (7%). Additionally, respondents highlighted the lack of opportunities for CVAD insertion in the subclavian vein, and 16% noted variability and scarcity of training resources and assessment frameworks in their respective ICUs.

**Table 4:** Free text responses presented thematically for the 44 responses.

Themes and number of responses n (%)	Experience <3 years	Experience ≥3 years
Lack of opportunities for CVAD insertion in subclavian vein and would want more opportunity 6 [9%])	- Lack of subclavian CVAD insertion teaching. - Have never seen subclavian CVAD being inserted. - Subclavian cannulation is increasingly underutilised due to over-reliance on ultrasound and over-estimating the risk of pneumothorax. - Less/no opportunities for subclavian lines, concerned that not getting enough opportunity of subclavian and would think that simulation would be helpful.	- As an experienced trainee, I have never had the opportunity to insert a subclavian line. Very few times when this is the most appropriate line placement for a patient as safer options (either IJ or femoral) are often available. However, I am concerned that I may become a consultant having never done one yet be called upon to do one. Some simulation regarding this site specifically may be helpful for trainees given it may be the only experience they will get during their training. - There MUST be feedback to QLD hospitals (especially tertiary Brisbane metro) that the teaching of subclavian lines is woeful. They must stop taking them from registrars so we can learn!
Advocate online learning (5 [11.4%])	- Advocate uniform online resource with short quiz to reinforce learning. - Prereading/guidelines would be helpful to review. - Education and awareness about complications and understanding of why we use different safety nets. - Watching video on CVC insertion is helpful. - Back the online learning/prereading especially for physiologically and anatomically difficult line insertions. - Recommends strongly for online learning from personal experience.	
Variability and scarcity resources/training/assessment in different ICUs (7 [15.9%])	- Lack of human resources to supervise delays the training of trainee. - No formal teaching at the regional ICU where the trainee works. - Variable quality and availability of teaching among various units. - Balancing realism of possibility of training at peripheral centre vs. expectation. - Lack of standardised assessment of competency. - Variable training between tertiary and metropolitan ICUs. - Less opportunities at smaller ICUs.	
Advocates targeted supervision for clinical and simulated scenarios (10 [22.7%])	- Advocate different level of supervision and assessment methods for different trainees in regard to their experience. - Advocates supervision compared to online education. - Supervision is more valuable tool for the experienced trainee if they feel rusty at times while insertion CVADs. - Recommends graduated supervision form close to distant. - Recommends separate supervision for difficult CVAD insertions. - Recommends focussing supervision on certain steps such as asepsis/confirming the guidewire etc. - Recommends supervising the whole procedure, getting the line prepared and letting the trainee to see someone else performing the insertion to learn more objectively. - Advocates specific supervision points for CVAD insertions at different sites and for Vascath insertion. - Advocated simulation for difficult scenarios. - CVAD insertion training with ultrasound guidance via simulation is very useful.	
Would like landmark technique to be taught (2 [4.5%])	- Landmark technique should be taught to enable trainees to insert line where US may not be useful, I E emphysema. - Promote teaching on landmark technique.	
Advocates US training with specific learning objectives (5 [11.4%])	- Promote US for training - Promote US training especially for new ICU trainee. - Advocates more hands on us practice for lack of coordination with the ultrasound/needle tip for novice trainee. - Advocate us training and US use for all CVADs. - Recommends formal US training and accreditation. - Emphasise on the US training as an important part of CVAD training to ensure safe insertion and avoid skipping important steps of US- needle coordination	
Advocated for checklist for supervisors and trainees (3 [6.8%])	- Promote checklist, - Identifies lack of the checklist for supervisors at the various ICUs where the participant has worked and recommends checklist. - Recommends checklist to avoid supervisor's bias and reduce variability.	
Recommends ongoing practice and assessment for maintenance of competency (3 [6.8%])	- Advocates on a greater number of CVAD insertion for novice trainee and maintenance of competency by performing CVAD insertion regularly. - Recommends regular work-based assessment and compare the performance against accepted standards. - Advocates continuous practice for maintaining competency.	
Miscellaneous (5 [11.4%])	- Difficulty to be supervised “in hours” prior to being the reg on nights and performing the procedure. - Advocates that once trainee is deemed competent to perform independent CVAD insertion procedure, the fellow of CICM can sign the CICM WCA. - A reference guide regarding best practice would be very useful. - Recommends delayed learning of subclavian after being confident of inserting in internal jugular and femoral veins. - Criticism of the survey especially as participant felt inability to find appropriate options of answers in the survey and had written answers in comments.	

CICM – College of Intensive Care Medicine, CVAD – central venous access device, ICU – intensive care unit.

These findings highlight key areas for improvement in CVAD training and accreditation, including the need for structured multimodal training, standardised competency assessments, and enhanced accessibility to training resources across different healthcare settings.

## 4 Discussion

### 4.1 Key findings

The study highlights the variability in CVAD training across different ICU settings and experience levels. Many trainees, particularly those with less than three years of experience, reported receiving no formal training. Additionally, competency assessments for CVAD insertion were rare. Trainees found ultrasound coordination, guidewire, and catheter manipulation particularly challenging in their early years. The use of manometry and transducers for CVAD position confirmation was also low. Trainers recommended integrating ultrasound into competency checks and increasing targeted supervision for both clinical and simulated procedures.

### 4.2 Training and accreditation for CVAD insertion skills

Comprehensive education, including competency assessments, has been shown to reduce complications.^3^ Despite evidence supporting periodic competency reassessment.^8^ This study underscores considerable variation in CVAD training across ICUs, consistent with findings from the CLIPER study.^2^ Notably, two-thirds of participants had not received training in the past year, with over 70% of less-experienced trainees having not undergone skill evaluations. With increasing trainee numbers, procedural exposure is at risk of dilution.^9^ Nearly half (46%) lacked multimodal training (combining online learning, ultrasound, simulation, and supervised practice), while 15% had no training at all. Supervised insertions without prior training, similar to the CLIPER study^2^ were common, especially in private and regional/rural ICUs. The absence of formalised training and credentialling contradicts best-practice recommendations, which advocate for structured teaching and competency-based assessments prior to unsupervised practice.^10^

Trainees expressed concerns over unclear training resources, highlighting the need for structured, easily applicable educational materials. The World Congress of Vascular Access (WoCoVA) recommends that training should encompass anatomy, ultrasound usage, sterile techniques, device selection, complication management, and competency assessments.^11^ Simulation-based training has been shown to improve learner performance in ultrasound use,^12^ sterile technique adherence,^13^ and first-pass success rates.^14^ However, patient outcomes are heterogeneous in regard to learner levels, types of simulation-based interventions, and comparative “traditional” training.^15^ While no studies directly compare the timing of simulation training and skill retention, supportive online modules may help trainees prepare prior to simulation sessions.^16^

The lack of standardised accreditation processes for CVAD insertion may contribute to inconsistent procedural competency among trainees and increased patient risk, especially because two-thirds of less-experience trainees reported that they were supervising junior colleagues. Furthermore, many supervisors did not use checklists (over two-thirds), and 30% of trainees reported inadequate feedback. Supervision by inexperienced practitioners may pose safety risks.^11^ Gonge et al. showed that supervision was varied when no direction about how to approach was available.^17^ Although, supervisor checklists may improve the clarity and efficiency of supervision, global rating scales completed by trained assessors remain the gold standard for evaluating procedural competence as supported by existing literature.^18^

### 4.3 Ultrasound use for CVAD insertion

Ultrasound significantly reduces complications during CVAD insertion^4^ and is recommended as a standard of care.^19^ We found that ultrasound use was inconsistently incorporated into formal training or assessment frameworks. Among trainees, 97% used the short-axis view, two-thirds assessed vein clotting, and 93% confirmed guidewire placement before dilation. Studies show the benefits of both short- and long-axis approaches, with one study showing short-axis plane leading to vein puncture with a shorter insertion time, better first-puncture success rate, and fewer complications,^20^ while another study demonstrated fewer posterior wall punctures with long-axis plane.^4^ The Society of Hospital Medicine recommends pre-procedural ultrasound assessment to evaluate vessel size, depth, and thrombosis risk.^21^ It was reassuring to note that a higher number of less-experienced trainees regularly assessed anatomical variations, vessel size, and needle tip positioning.

Real-time ultrasound significantly reduces mechanical complications and improves first-pass success. Tapan et al. demonstrated that intensivists in Australia and New Zealand use blood gas (35.6%) and ultrasound (62.1%) for CVAD confirmation.^2^ When compared to intensivists, most of the trainees used real-time ultrasound (96%) and blood gas analysis (79%) to confirm vein cannulation. The trainees also used post-procedure ultrasound (42%), chest X-ray (88%), transducer (37%), and manometry (14%) to confirm vein cannulation. In contrast, more experienced trainees relied less on blood gas confirmation.

### 4.4 Challenges in early CVAD training

The early stages of CVAD training are marked by distinct technical challenges. Two-thirds of trainees found ultrasound coordination challenging in their early years, particularly aligning the probe with the vein. Understanding ultrasound physics, image acquisition, and interpretation of 2D images representing 3D anatomical structures is essential to overcoming these difficulties.^3^ Guidewire and catheter manipulation were challenging for 32–39% of trainees in their initial months, with one-quarter struggling to position the CVAD tip correctly. These findings are consistent with prior studies indicating that early learners struggle with most procedural spatial orientation and real-time coordination under supervision.^22^ Standardised educational approaches combining supervision, simulation, and structured training improve skill acquisition and reduce complications.^23^ Another concerning finding was the reported lack of training in subclavian vein cannulation. Despite its continued relevance in clinical practice, especially when other sites are contraindicated or inaccessible, many trainees reported receiving little or no instruction or supervised experience in this technique. This omission may stem from institutional preferences for jugular or femoral access, but it creates a gap in the trainee's procedural repertoire and may impair their ability to make site-specific decisions in complex cases.

### 4.5 Trainee recommendations

Although responses were limited, the trainees emphasised the need for online and ultrasound training with clear objectives and targeted supervision in both clinical and simulated settings. Most suggested using ultrasound for competency assessment, while fewer endorsed simulation (17%) or online assessment for experienced trainees (19%). Previous recommendations suggest that training should include didactic sessions, simulated practice, supervised patient insertions, and regular competency assessments.^3^ Standardised checklists for supervisors and trainees were also recommended. A small proportion (9%) noted limited opportunities for subclavian vein insertions, likely due to site preferences among Australian intensivists.^2^ Additionally, 16% reported a lack of training resources in their ICUs. Despite resource limitations, structured education and standardised credentialing processes could enhance patient safety and procedural competency.^24^ Therefore, these findings should be interpreted as preliminary insights to guide future curriculum evaluation and redesign, which should ultimately be informed by educational theory, comprehensive needs assessment, and expert input.

### 4.6 Strengths and limitations

This is the largest survey to assess ICU trainees' CVAD training and practice across Australia and New Zealand, with responses from various experience levels and ICU types. Furthermore, the alignment of many findings with the CLIPER study^2^ enhances the validity of the results. However, the study is subject to inherent response and sampling bias, particularly as a disproportionate number of respondents may come from hospitals with more resources. Also, as participation was voluntary and responses were self-reported, there is a possibility that trainees with greater interest or confidence in procedural training were more likely to respond, and that recollections of training experiences may have been inaccurate or incomplete. The modest response rate, predominance of respondents from two Australian states, and the potential for selection, response, and recall biases limit the generalisability of these findings. Additionally, the fixed-response design and certain question wording and formatting may have restricted some participants’ ability to fully express their experiences.

### 4.7 Future directions

Findings from this and previous studies provide a strong foundation for developing a standardised, competency-based training and accreditation framework for CVAD insertion. This data will help shape structured CVAD education programs, including online modules, standardised simulation, ultrasound training objectives, and supervised practice guidelines for adoption across ICUs. These findings represent trainee perceptions rather than direct measures of training effectiveness and should be interpreted within this context. Future research using a mixed-methods design, integrating qualitative interviews and input from education leads, could provide a more comprehensive understanding of the factors influencing CVAD training quality and consistency across sites. Furthermore, future research should also assess the effectiveness of uniform training and accreditation programs by evaluating trainee-oriented outcomes.

## 5 Conclusion

The study reveals significant variability in CVAD training across ICUs and experience levels. Many trainees lacked the multimodal training they recommended, such as supervised practice, simulation, ultrasound training with defined objectives, checklists, and online modules. Findings align with the earlier CLIPER study, where ICU consultants were surveyed. Together, these studies, based on reported trainee perceptions, can inform needs assessments and support the development of standardised policies and curricula to reduce variability and enhance care quality.

## Funding

This research received no specific grant from any funding agency in the public, commercial, or not-for-profit sectors.

## CRediT authorship contribution statement

**Tapan Parikh:** Conceptualization, Methodology, Data Curation, Formal analysis, Writing - Original Draft, Visualisation **Wisam Al Bassam:** Conceptualization, Methodology **Yahya Shehabi:** Methodology **Deepak Bhonagiri:** Conceptualization, Methodology **Tim Leong:** Methodology **Adrian Pakavakis:** Methodology **Brendan Murfin:** Methodology **Ashwin Subramaniam:** Conceptualization, Methodology, Formal analysis, Writing - Original Draft.

## Conflicts of interest

The authors declare the following financial interests/personal relationships which may be considered as potential competing interests: Dr Tapan Parikh reports administrative support was provided by College of Intensive Care Medicine of Australia and New Zealand. Dr Tapan Parikh reports administrative support was provided by Australian and New Zealand Intensive Care Society. If there are other authors, they declare that they have no known competing financial interests or personal relationships that could have appeared to influence the work reported in this paper.
