# Supplementary material for: Current practice, training and skill assessment of central venous access device insertion: Perspectives of intensive care trainees in adult intensive care units across Australia and New Zealand and their recommendations for improvement: Central Venous Lines Insertion: Practice, Education, Recommendation 2.0 (CLIPER 2.0)
Source: Crit Care Resusc. 2026 Feb 19;28(1):100159. doi: 10.1016/j.ccrj.2025.100159 (PMC12936742; doi:10.1016/j.ccrj.2025.100159)
Supplement: Multimedia component 1 [file mmc1.zip › CCRJ-D-25-00163/Supplementary material CLIPER 2.docx]

**Supplementary Figure 1:** Training experience based on hospital type.


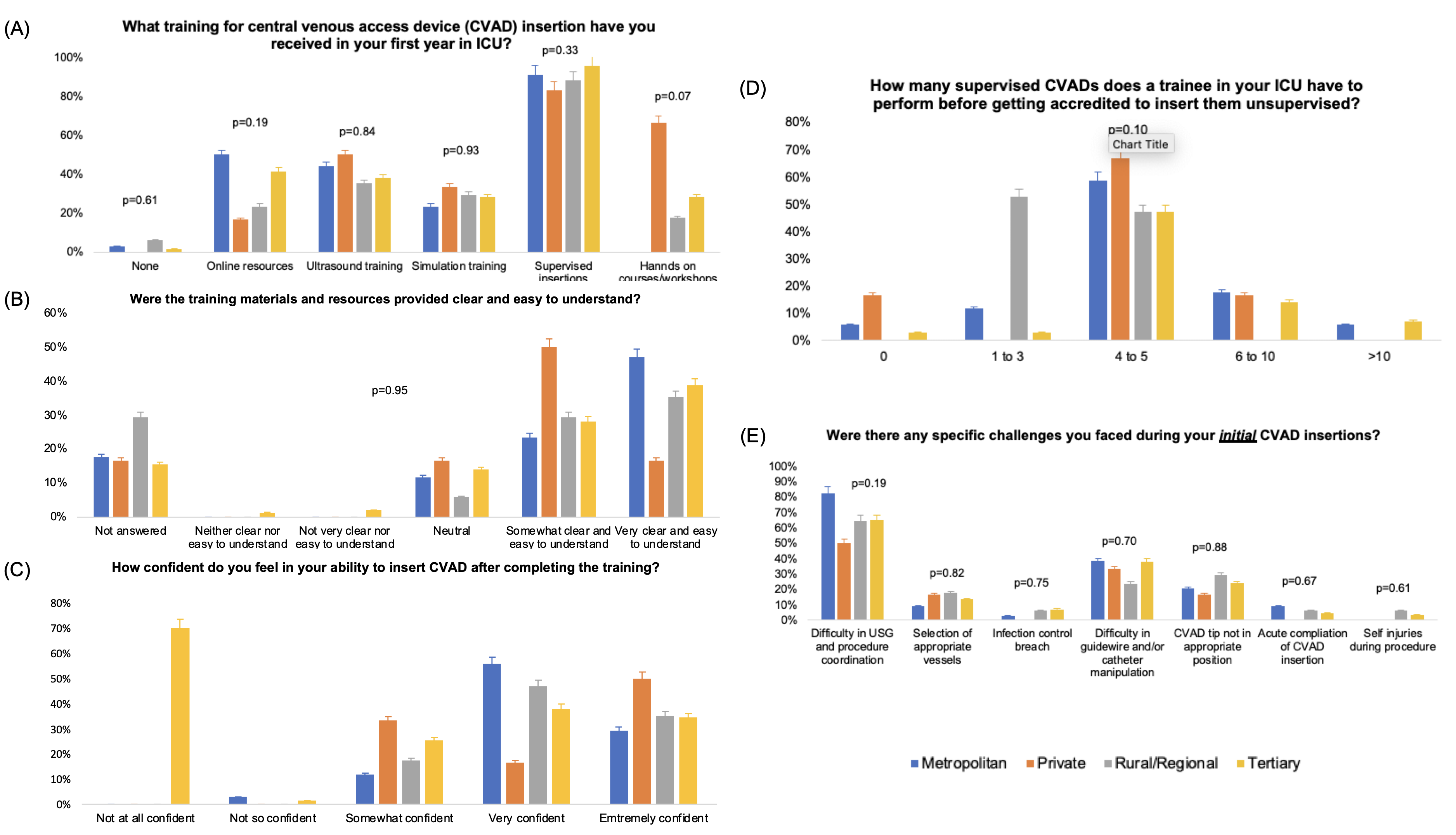


**Supplementary Figure 2:**


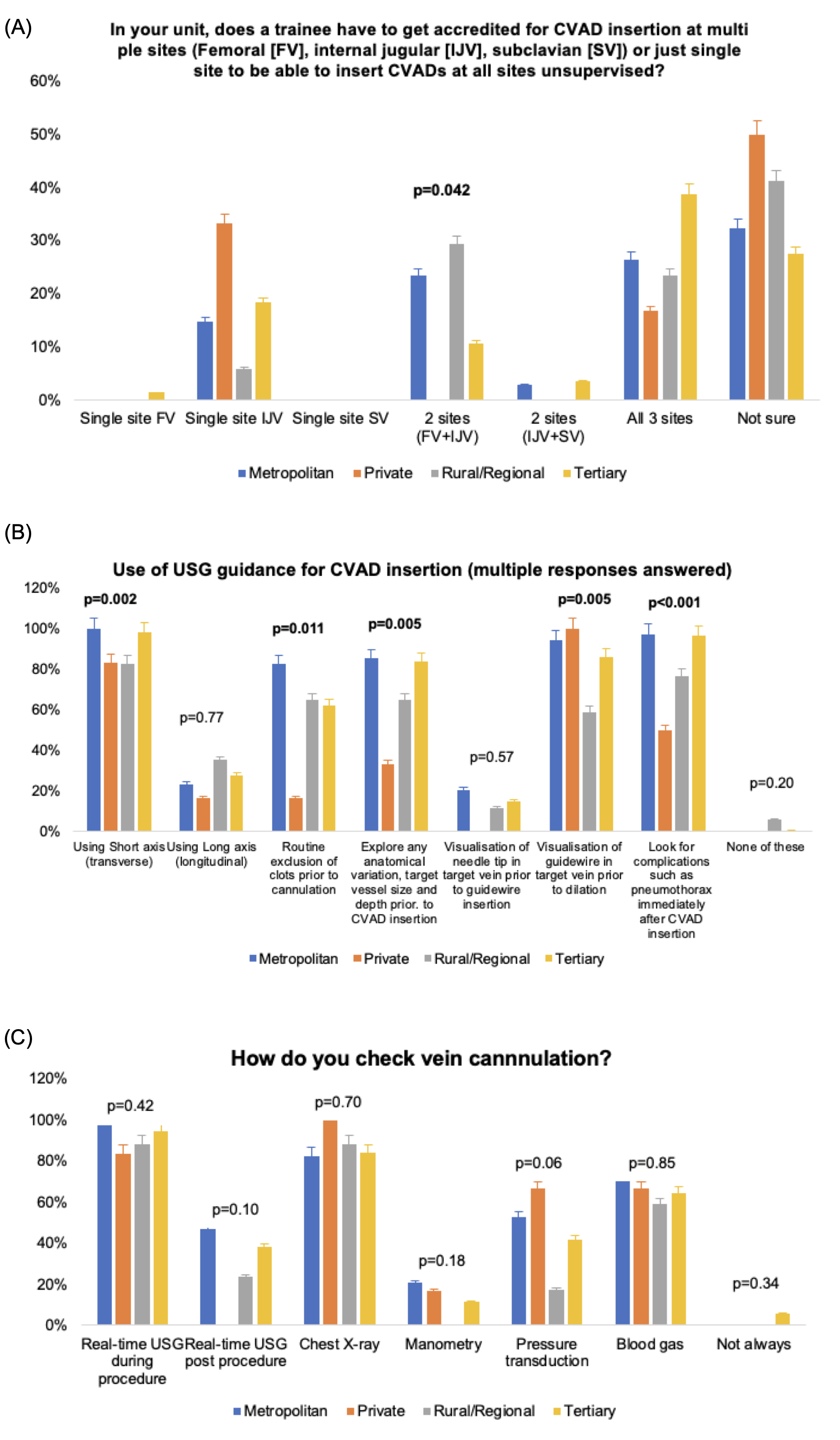


**Supplementary Figure 3:** Training experience based on jurisdiction.


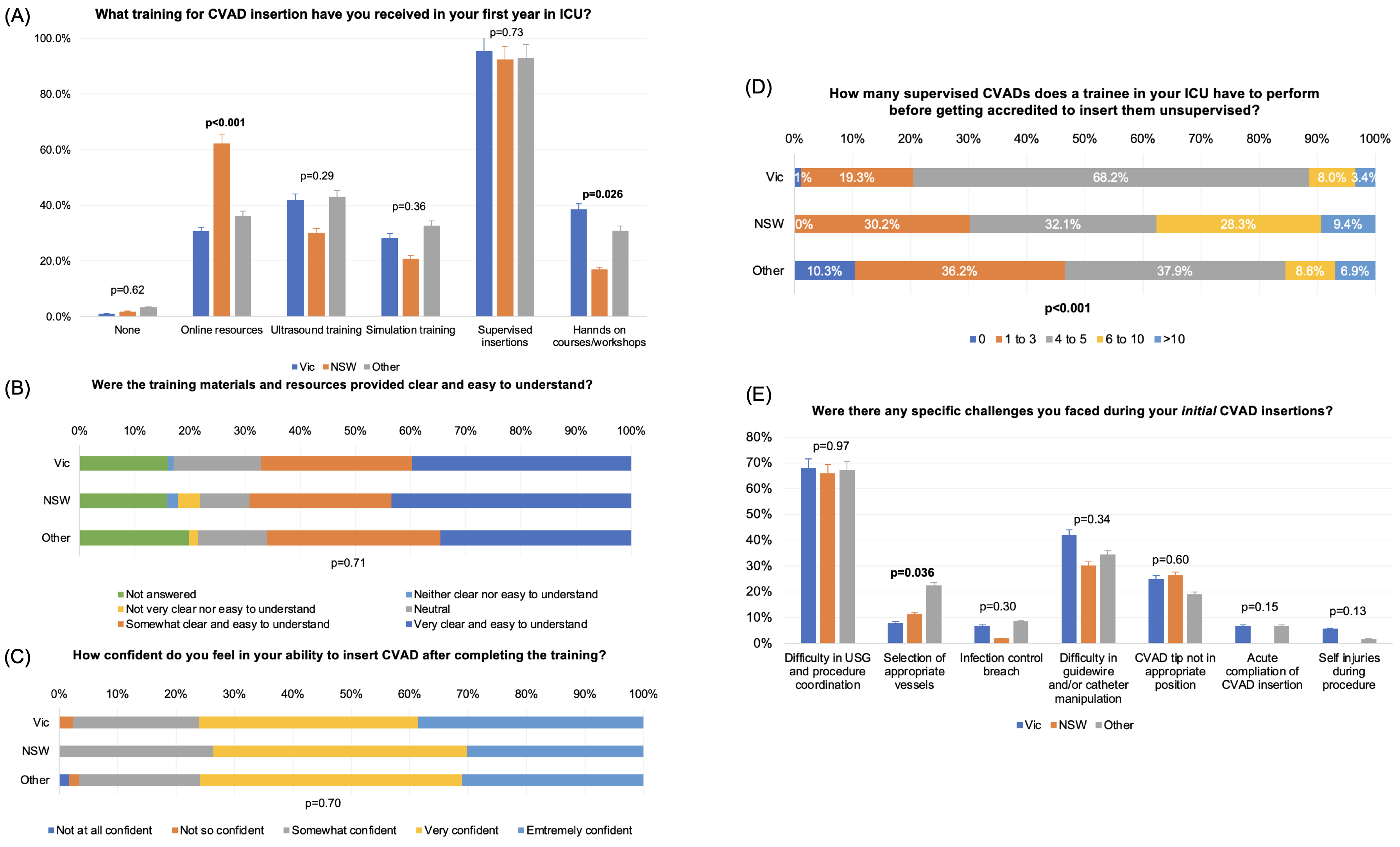


**Supplementary Figure 4:**


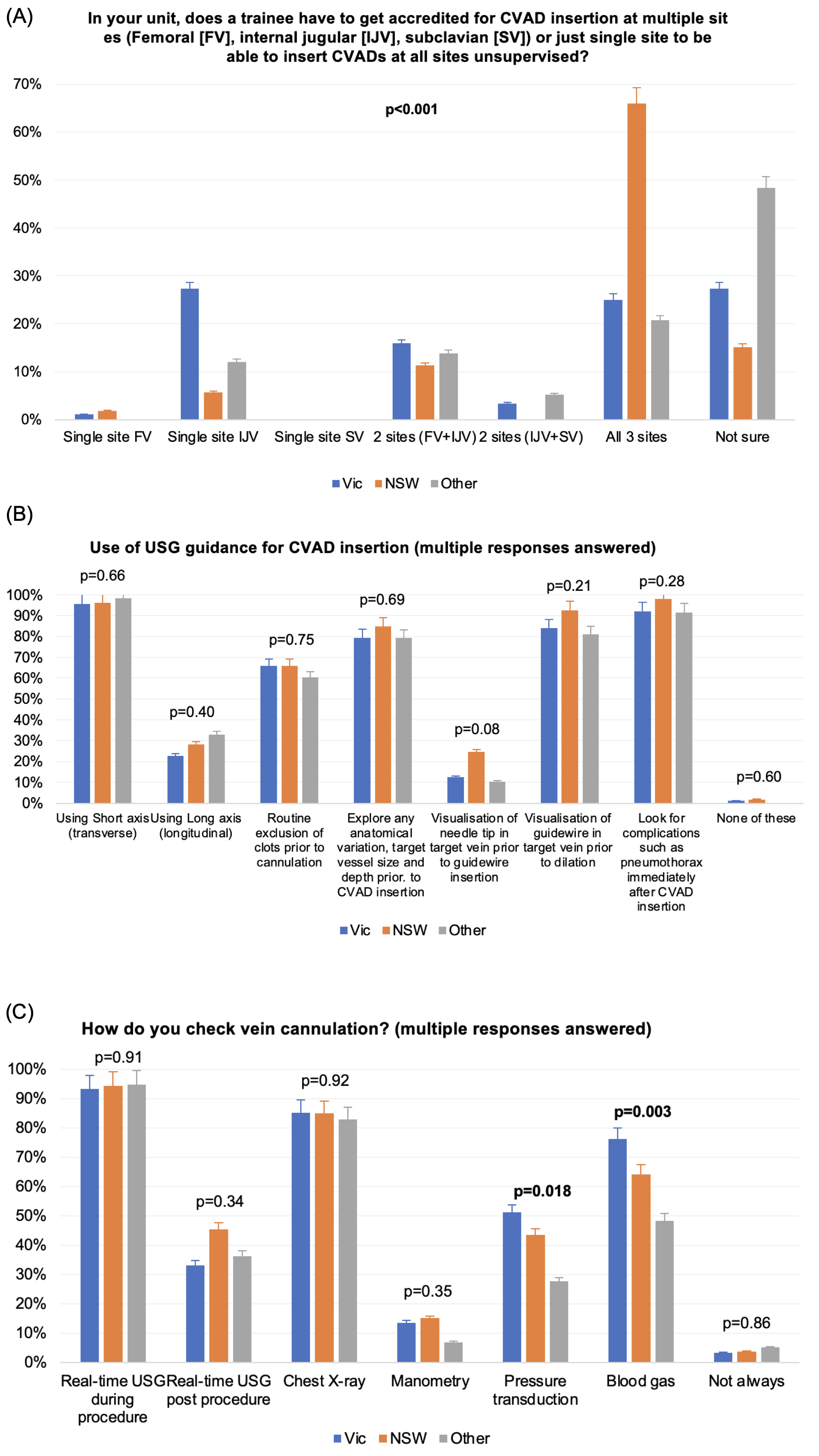


**Supplementary Table 1:** Baseline characteristics based on hospital type.

| **Table 1: Demographics** | **Metropolitan** | **Private** | **Rural/ Regional** | **Tertiary** | **p-value** |
| --- | --- | --- | --- | --- | --- |
| **Age** |  |  |  |  | 0.97 |
| - <25 years | 0 (0) | 0 (0) | 0 (0) | 2 (1.4%) |  |
| - 25-30 years | 12 (35.3%) | 1 (16.7%) | 5 (29.4%) | 41 (28.9%) |  |
| - 30-40 years | 18 (52.9%) | 4 (66.7%) | 9 (52.9%) | 83 (58.5%) |  |
| - >40 years | 4 (11.8%) | 1 (16.7%) | 3 (17.7%) | 16 (11.3%) |  |
| **Gender** |  |  |  |  | **0.004** |
| - Male | 25 (73.5%) | 6 (100%) | 10 (58.8%) | 78 (54.9%) |  |
| - Female | 9 (26.5%) | 0 (0) | 6 (35.3%) | 64 (45.1%) |  |
| - Non-binary | 0 (0) | 0 (0) | 1 (5.9%) | 0 (0) |  |
| **Country of work** |  |  |  |  | 0.25 |
| **Australia** | **34 (100%)** | **6 (100%)** | **132 (93.0%)** |  |  |
| - Australian capital territory | 0 (0) | 0 (0) | 0 (0) | 2 (1.4%) |  |
| - New South Wales | 6 (17.6%) | 0 (0) | 4 (23.5%) | 43 (30.3%) |  |
| - Northern territory | 1 (2.9%) | 0 (0) | 0 (0) | 0 (0) |  |
| - Queensland | 6 (17.6%) | 2 (33.3%) | 4 (23.5%) | 11 (7.7%) |  |
| - South Australia | 1 (2.9%) | 0 (0) | 0 (0) | 8 (5.6%) |  |
| - Tasmania | 0 (0) | 0 (0) | 1 (5.9%) | 4 (2.8%) |  |
| - Victoria | 20 (58.8%) | 4 (66.7%) | 5 (29.4%) | 59 (41.5%) |  |
| - Western Australia | 0 (0) | 0 (0) | 1 (5.9%) | 5 (3.5%) |  |
| **New Zealand** | **0 (0)** | **0 (0)** | **10 (7.0%)** |  |  |
| - North Island | 1 (2.9%) | 0 (0) | 0 (0) | 0 (0) |  |
| - South Island | 0 (0) | 0 (0) | 0 (0) | 6 (4.2%) |  |
| **Other (Singapore)** | **0 (0)** | **0 (0)** | **1 (5.9%)** | **0 (0)** |  |
| **Experience** |  |  |  |  | **0.019** |
| - <3 years | 15 (44.1%) | 1 (16.7%) | 12 (70.6%) | 49 (34.5%) |  |
| - ≥3 years | 19 (55.9%) | 5 (83.3%) | 5 (29.4%) | 93 (65.5%) |  |
| **>12 months Experience in Emergency Department and/or Anaesthetics** | 26 (76.5%) | 5 (83.3%) | 9 (52.9%) | 105 (73.9%) | 0.26 |
| **Type of Trainee** |  |  |  |  | **0.001** |
| - Pre-primary exam | 15 (44.1%) | 2 (33.3%) | 5 (29.4%) | 52 (36.6%) |  |
| - Post-primary exam | 7 (20.6%) | 1 (16.7%) | 3 (17.6%) | 58 (40.8%) |  |
| - Post fellowship exam | 5 (14.7%) | 1 (16.7%) | 1 (5.9%) | 22 (15.5%) |  |
| - Unaccredited ICU registrar/CMO | 7 (20.5%) | 2 (33.4%) | 8 (47.0%) | 10 (7.0%) |  |
| **Years of experience** |  |  |  |  | 0.07 |
| - 0 to 3 months | 1 (2.9%) | 2 (2.6%) | 0 (0) | 1 (0.7%) |  |
| - 3 to 6 months | 0 (0) | 0 (0) | 0 (0) | 0 (0) |  |
| - 6 months to 1 year | 3 (8.8%) | 1 (16.7%) | 4 (23.5%) | 8 (5.6%) |  |
| - 1 to 3 years | 11 (32.4%) | 0 (0) | 8 (47.1%) | 40 (28.2%) |  |
| - 3 to 5 years | 7 (20.6%) | 0 (0) | 3 (17.6%) | 41 (28.9%) |  |
| - 5 to 10 years | 9 (26.5%) | 4 (66.7%) | 0 (0) | 39 (27.5%) |  |
| - >10 years | 3 (8.8%) | 1 (16.7%) | 2 (11.8%) | 13 (9.2%) |  |
| **Working continuously in ICU past 12 months** | 19 (55.9%) | 3 (50.0%) | 11 (64.7%) | 96 (67.6%) | 0.52 |
| **Commenced work in ICU within <12 months** |  |  |  |  | 0.44 |
| - ≤6 months (n=39) | 8 (47.1%) | 3 (100%) | 4 (44.4%) | 24 (32.0%) |  |
| - 6-12 months (n= | 9 (52.9%) | 0 (0) | 5 (55.6%) | 51 (68.0%) |  |
| CMO – career medical officer, ICU – intensive care unit | | | | | |

**Supplementary Table 2:** Training experience and recommendations for training and accreditation based on hospital type.

|  |  | **Metropolitan** | **Private** | **Rural/ Regional** | **Tertiary** | **p-value** |
| --- | --- | --- | --- | --- | --- | --- |
| **Training Experience** |  |  |  |  |  |  |
| Training received for CVAD insertion in the last 12 months? | No | 20 (58.8%) | 5 (83.3%) | 12 (70.6%) | 97 (68.3%) | 0.58 |
|  | Yes | 14 (41.2%) | 1 (16.7%) | 5 (29.4%) | 45 (31.7%) |  |
| Does your ICU currently implement structured training programs incorporating multimodal methods, such as online and paper-based training, simulation, ultrasonography, and supervised practice? | I don’t know | 6 (17.6%) | 0 (0) | 1 (5.9%) | 20 (14.1%) | **<0.001** |
|  | No | 8 (23.5%) | 5 (83.3%) | 13 (76.5%) | 38 (26.8%) |  |
|  | Yes | 20 (58.8%) | 1 (16.7%) | 3 (17.6%) | 84 (59.2%) |  |
| If the answer was ‘No’ or ‘I don’t know’ to the previous question, does your ICU have individual formal USG/simulation training, or supervised practice? | Neither | 2 (5.9%) | 4 (66.7%) | 4 (23.5%) | 9 (6.3%) | **0.009** |
|  | Only simulation | 0 (0) | 0 (0) | 0 (0) | 1 (0.7%) |  |
|  | Only supervision | 8 (23.5%) | 0 (0) | 4 (23.5%) | 31 (21.8%) |  |
|  | Only USG | 1 (2.9%) | 0 (0) | 1 (5.9%) | 3 (2.1%) |  |
|  | Simulation+ supervision | 1 (2.9%) | 0 (0) | 2 (11.8%) | 12 (8.5%) |  |
|  | USG+ Simulation | 0 (0) | 0 (0) | 0 (0) | 3 (2.1%) |  |
|  | USG+ supervision | 10 (29.4%) | 1 (16.7%) | 4 (23.5%) | 23 (16.2%) |  |
| Do you use a **checklist** for CVAD insertion? | No | 19 (55.9%) | 3 (50.0%) | 11 (64.7%) | 80 (56.3%) | 0.96 |
|  | Not sure | 1 (2.9%) | 0 (0) | 1 (5.9%) | 7 (4.9%) |  |
|  | Yes | 14 (41.2%) | 3 (50.0%) | 5 (29.4%) | 55 (38.7%) |  |
| Does your supervisor for CVAD insertion use a **checklist** to assess you? | No | 18 (52.9%) | 4 (66.7%) | 9 (52.9%) | 74 (52.1%) | 0.85 |
|  | Not sure | 5 (14.7%) | 1 (16.7%) | 5 (29.4%) | 28 (19.7%) |  |
|  | Yes | 11 (32.4%) | 1 (16.7%) | 3 (17.6%) | 40 (28.2%) |  |
| Do you feel that you were provided reasonable feedback by your supervisor who assessed your CVAD insertion skills? | No | 4 (11.8%) | 2 (33.3%) | 4 (23.5%) | 29 (20.4%) | 0.60 |
|  | Not sure | 4 (11.8%) | 0 (0) | 0 (0) | 14 (9.9%) |  |
|  | Yes | 26 (76.5%) | 4 (66.7%) | 13 (76.5%) | 99 (69.7%) |  |
| Have you undergone an assessment of check for competency of your CVAD insertion skills in the last 12 months | No | 22 (64.7%) | 6 (100%) | 15 (88.2%) | 110 (77.5%) | 0.12 |
|  | Yes | 12 (35.3%) | 0 (0) | 2 (11.8%) | 32 (22.5%) |  |
| Do you train junior doctors in inserting CVADs? | No | 2 (5.9%) | 2 (33.3%) | 4 (23.5%) | 25 (17.6%) | 0.53 |
|  | Yes | 32 (94.1%) | 4 (66.7%) | 13 (76.5%) | 117 (82.4%) |  |
| **Training Accreditation** |  |  |  |  |  |  |
| How many CVADs should be supervised for novice trainees for accreditation in ICU? | <5 | 9 (26.5%) | 1 (16.7%) | 9 (52.9%) | 44 (31.0%) | 0.50 |
|  | 6-10 | 18 (52.9%) | 4 (66.7%) | 7 (41.2%) | 75 (52.8%) |  |
|  | >10 | 7 (20.6%) | 1 (16.7%) | 1 (5.9%) | 23 (16.2%) |  |
| How many CVAD sites should be supervised for novice trainees for accreditation in ICU? | Single site | 0 (0) | 0 (0) | 0 (0) | 3 (2.1%) | 0.55 |
|  | Multi-site | 22 (64.7%) | 2 (33.3%) | 8 (47.1%) | 87 (61.3%) |  |
|  | Not answered | 12 (35.3%) | 4 (66.7%) | 9 (52.9%) | 52 (36.6%) |  |
| How should the competency for maintenance of CVAD insertion skills be assessed? | Supervision of CVAD insertion + USG technique | 30 (88.2%) | 5 (83.3%) | 15 (88.2%) | 121 (85.2%) | 0.96 |
|  | Assessment by Simulation | 7 (20.6%) | 2 (33.3%) | 1 (5.9%) | 16.9%) | 0.40 |
|  | Online Assessment only | 8 (23.5%) | 1 (16.7%) | 1 (5.9%) | 27 (19.0%) | 0.50 |
| CVAD – central venous access device, ICU – intensive care unit, USG – ultrasound guidance | | | | | | |

**Supplementary Table 3:** Baseline characteristics based on jurisdiction.

p=0.06

p=0.63

| **Demographics** | **Victoria** | **New South Wales** | **Other Australian States, New Zealand** | **p-value** |
| --- | --- | --- | --- | --- |
| **Number** | 88 | 53 | 58 | - |
| **Age** |  |  |  | 0.67 |
| - <25 years | 1 (1.1%) | 1 (1.9%) | 0 (0) |  |
| - 25-30 years | 25 (28.4%) | 19 (35.8%) | 15 (25.9%) |  |
| - 30-40 years | 49 (55.7%) | 29 (54.7%) | 36 (62.1%) |  |
| - >40 years | 13 (14.8%) | 4 (7.6%) | 7 (12.1%) |  |
| **Gender** |  |  |  | 0.32 |
| - Male | 58 (65.9%) | 24 (45.3%) | 32 (55.2%) |  |
| - Female | 30 (34.1%) | 24 (45.3%) | 25 (43.1%) |  |
| - Non-binary | 0 (0) | 0 (0) | 1 (1.7%) |  |
| **Hospital type** |  |  |  | 0.17 |
| - Tertiary | 59 (67.0%) | 43 (81.1%) | 40 (69.0%) |  |
| - Metropolitan | 20 (22.7%) | 6 (11.3%) | 8 (13.8%) |  |
| - Rural/Regional | 5 (5.7%) | 4 (7.5%) | 8 (13.8%) |  |
| - Private | 4 (4.5%) | 0 (0) | 2 (3.4%) |  |
| **>12 months Experience in Emergency Department and/or Anaesthetics** | 64 (72.7%) | 43 (81.1%) | 38 (65.5%) | 0.18 |
| **Type of Trainee** |  |  |  | 0.95 |
| - Pre-primary exam | 36 (40.9%) | 19 (35.8%) | 19 (32.8%) |  |
| - Post-primary exam | 26 (29.5%) | 21 (39.6%) | 22 (37.9%) |  |
| - Post fellowship exam | 14 (15.9%) | 7 (13.2%) | 8 (13.8%) |  |
| - Unaccredited ICU registrar/CMO | 4 (5.4%) | 6 (11.3%) | 9 (15.5%) |  |
| **Years of experience** |  |  |  | 0.44 |
| - 0 to 3 months | 2 (12.3%) | 0 (0) | 0 (0) |  |
| - 3 to 6 months | 0 (0) | 0 (0) | 0 (0) |  |
| - 6 months to 1 year | 7 (8.0%) | 4 (7.5%) | 5 (8.6%) |  |
| - 1 to 3 years | 22 (25.0%) | 22 (41.5%) | 15 (25.9%) |  |
| - 3 to 5 years | 27 (30.7%) | 9 (17.0%) | 15 (25.9%) |  |
| - 5 to 10 years | 21 (23.9%) | 15 (28.3%) | 16 (27.6%) |  |
| - >10 years | 9 (10.2%) | 3 (5.7%) | 7 (12.1%) |  |
| **Working continuously in ICU past 12 months** | 61 (69.3%) | 33 (62.3%) | 35 (60.3%) | 0.49 |
| **Commenced work in ICU within <12 months** |  |  |  | 0.83 |
| - ≤6 months | 15 (39.5%) | 13 (48.1%) | 11 (44.0%) |  |
| - 6-12 months | 23 (60.5%) | 14 (51.9%) | 14 (66.0%) |  |
| CMO – career medical officer, ICU – intensive care unit | | | | |

**Supplementary Table 4:** Training experience and recommendations for training and accreditation based on jurisdiction.

|  |  | **Victoria** | **New South Wales** | **Other Australian States, New Zealand** | **p-value** |
| --- | --- | --- | --- | --- | --- |
| **Training Experience** |  |  |  |  |  |
| No training received for CVAD insertion in the last 12 months? | No | 65 (73.9%) | 29 (54.7%) | 40 (69.0%) | 0.06 |
|  | Yes | 23 (26.1%) | 24 (45.3%) | 18 (31.0%) |  |
| Does your current ICU have structured training that includes combinations of multimodal methods such as online/paper-based training, simulation, USG, and supervised practice? | I don’t know | 11 (12.5%) | 8 (15.1%) | 8 (13.8%) | **0.031** |
|  | No | 27 (30.7%) | 10 (18.9%) | 27 (46.6%) |  |
|  | Yes | 50 (56.8%) | 35 (66.0%) | 23 (39.7%) |  |
| If the answer was ‘No’ or ‘I don’t know’ to the previous question, does your ICU have individual formal USG/simulation training, or supervised practice? | Neither | 8 (9.1%) | 4 (7.5%) | 7 (12.1%) | 0.36 |
|  | Only simulation | 0 (0) | 1 (1.9%) | 0 (0) |  |
|  | Only supervision | 43 (34.6%) | 24 (46.2%) | 19 (26.4%) |  |
|  | Only USG | 1 (1.1%) | 0 (0) | 4 (6.9%) |  |
|  | Simulation+ supervision | 8 (9.1%) | 3 (5.7%) | 4 (6.9%) |  |
|  | USG+ Simulation | 1 (1.1%) | 0 (0) | 2 (3.4%) |  |
|  | USG+ supervision | 17 (19.3%) | 10 (18.9%) | 11 (19.0%) |  |
| Do you use a **checklist** for CVAD insertion? | No | 47 (53.4%) | 27 (50.9%) | 39 (67.2%) | 0.36 |
|  | Not sure | 5 (5.7%) | 3 (5.7%) | 3 (1.7%) |  |
|  | Yes | 36 (40.9%) | 23 (43.4%) | 18 (31.0%) |  |
| Does your supervisor for CVAD insertion use a **checklist** to assess you? | No | 40 (45.5%) | 31 (58.5%) | 34 (58.6%) | 0.46 |
|  | Not sure | 20 (22.7%) | 10 (18.9%) | 9 (15.5%) |  |
|  | Yes | 28 (31.8%) | 12 (22.6%) | 15 (25.9%) |  |
| Do you feel that you were provided reasonable feedback by your supervisor who assessed your CVAD insertion skills? | No | 16 (18.2%) | 10 (18.9%) | 13 (22.4%) | 0.91 |
|  | Not sure | 8 (9.1%) | 6 (11.3%) | 4 (6.9%) |  |
|  | Yes | 64 (72.7%) | 37 (69.8%) | 41 (70.7%) |  |
| Have you undergone an assessment of check for competency of your CVAD insertion skills in the last 12 months | No | 68 (77.3%) | 40 (75.5%) | 45 (77.6%) | 0.96 |
|  | Yes | 20 (22.7%) | 13 (24.5%) | 13 (22.4%) |  |
| Do you train junior doctors in inserting CVADs? | No | 13 (14.7%) | 9 (17.0%) | 11 (17.0%) | 0.74 |
|  | Yes | 75 (85.3%) | 44 (83.0%) | 47 (81.0%) |  |
| **Trainee accreditation** |  |  |  |  |  |
| How many CVADs should be supervised for novice trainees for accreditation in ICU? | <5 | 25 (28.4%) | 15 (28.3%) | 23 (39.7%) | 0.42 |
|  | 6-10 | 50 (56.8%) | 30 (56.6%) | 24 (41.4%) |  |
|  | >10 | 13 (14.8%) | 8 (15.1%) | 11 (19.0%) |  |
| How many CVAD sites should be supervised for novice trainees for accreditation in ICU? | Single site | 0 (0) | 2 (3.8%) | 1 (1.7%) | 0.37 |
|  | Multi-site | 50 (56.8%) | 32 (60.4%) | 37 (63.8%) |  |
|  | Not answered | 38 (43.2%) | 19 (35.8%) | 20 (34.5%) |  |
| How should the competency for maintenance of CVAD insertion skills be assessed? | Supervision of CVAD insertion + USG technique | 73 (83.0%) | 45 (84.9%) | 53 (91.4%) | 0.35 |
|  | Assessment by Simulation | 20 (22.7%) | 5 (9.4%) | 9 (15.5%) | 0.12 |
|  | Online Assessment only | 18 (20.5%) | 11 (20.8%) | 8 (13.8%) | 0.54 |
| CVAD – central venous access device, ICU – intensive care unit, USG – ultrasound guidance | | | | | |
